# Self-referenced photonic chip soliton Kerr frequency comb

**DOI:** 10.1038/lsa.2016.202

**Published:** 2017-01-13

**Authors:** Victor Brasch, Erwan Lucas, John D Jost, Michael Geiselmann, Tobias J Kippenberg

**Affiliations:** École Polytechnique Fédérale de Lausanne (EPFL) – IPHYS, Lausanne CH-1015, Switzerland

**Keywords:** frequency combs, integrated optics, Kerr frequency combs, metrology, microresonator frequency combs, nonlinear optics

## Abstract

Self-referencing turns pulsed laser systems into self-referenced frequency combs. Such frequency combs allow counting of optical frequencies and have a wide range of applications. The required optical bandwidth to implement self-referencing is typically obtained via nonlinear broadening in optical fibers. Recent advances in the field of Kerr frequency combs have provided a path toward the development of compact frequency comb sources that provide broadband frequency combs, exhibit microwave repetition rates and are compatible with on-chip photonic integration. These devices have the potential to significantly expand the use of frequency combs. Yet to date, self-referencing of such Kerr frequency combs has only been attained by applying conventional, fiber-based broadening techniques. Here we demonstrate external broadening-free self-referencing of a Kerr frequency comb. An optical spectrum spanning two-thirds of an octave is directly synthesized from a continuous wave laser-driven silicon nitride microresonator using temporal dissipative Kerr soliton formation and soliton Cherenkov radiation. Using this coherent bandwidth and two continuous wave transfer lasers in a 2*f–*3*f* self-referencing scheme, we are able to detect the offset frequency of the soliton Kerr frequency comb. By stabilizing the repetition rate to a radio frequency reference, the self-referenced frequency comb is used to count and track the continuous wave pump laser’s frequency. This work demonstrates the principal ability of soliton Kerr frequency combs to provide microwave-to-optical clockworks on a chip.

## Introduction

Nonlinear spectral broadening in optical fibers via supercontinuum generation^1, 2, 3^ can provide an octave of coherent optical bandwidth from pulsed lasers^4^. This discovery has been an essential step in realizing self-referencing schemes, which enable measuring the carrier envelope offset frequency of a frequency comb^5, 6^. By measuring the offset frequency (*f*_CEO_) and the repetition rate (*f*_rep_), the optical frequencies of all the comb lines can be precisely determined via the relation *ν*_*n*_=*f*_CEO_+*nf*_rep_, where *n* designates the respective comb line. This relation establishes a phase coherent link from the radio frequency (RF) to the optical domain and provides a ‘clockwork’ that enables counting of optical frequencies^4, 7^ or, in the reverse direction, the synthesis of optical frequencies from RFs^8^. These properties have made self-referenced frequency combs versatile precision tools for many applications such as optical atomic clocks^9^, spectroscopy^10^ and low-noise microwave generation^11^.

The discovery of Kerr frequency comb generation in optical microresonators^12, 13^, also known as microresonator frequency combs, has triggered substantial research efforts toward the development of compact frequency comb sources with repetition rates in the microwave regime (>10 GHz) and spectral operation from the near-infrared^14^ to the mid-infrared^15, 16^, which are fully compatible with on-chip photonic integration^17^. In the context of frequency metrology, one advantage of Kerr frequency combs is that due to their high repetition rates the line spacing is usually sufficiently large to be resolvable on lower resolution grating-based optical spectrometers. This greatly simplifies applications where the knowledge of the line number *n* is required^10^ or where the frequency comb is used to calibrate a spectrometer^18^. Kerr frequency combs have the potential to significantly extend the utility and range of applications of frequency combs by reducing size, complexity and costs, and they have already been successfully employed for a range of applications including coherent terabit communications^19^, atomic clocks^20^ and optical arbitrary waveform generation^21^.

The recent observation of Kerr frequency combs generated via dissipative temporal soliton formation^22, 23^ has been a pivotal development. Dissipative solitons in microresonators provide a reliable path toward fully coherent comb operation as well as access to femtosecond optical pulses at microwave repetition rates. Such dissipative Kerr solitons rely on the double balance of parametric gain and cavity loss, as well as of Kerr nonlinearity and dispersion^22, 23, 24, 25^ and have been generated in a number of microresonator platforms to date^23, 26, 27, 28, 29^.

Dissipative Kerr solitons in optical microresonators also provide a route to synthesize spectra that are sufficiently broad for self-referencing without the need of external broadening similar to Ti:sapphire lasers^30, 31^ and in contrast to previous demonstrations of self-referenced Kerr frequency combs that relied on both external amplification and broadening stages^32, 33^. Superseding these stages makes further on-chip integration of self-referenced Kerr frequency comb sources possible which could enable the realization of a fully chip-scale RF to optical link. The required optical bandwidth for self-referencing is achieved via the large cavity enhancement along with dispersion engineering^34^ in photonic chip-based silicon nitride (Si_3_N_4_) microresonators. This allows the generation of solitons for which the Raman effect^28, 35, 36^ and higher-order dispersion effects such as soliton Cherenkov radiation^37, 38^ (a process related to third-order dispersion and also known as dispersive wave emission) become relevant. Recent results have shown that the generation of coherent spectra spanning two-thirds of an octave is possible using dispersive wave emission^27^.

## Materials and methods

**Figure 1:**
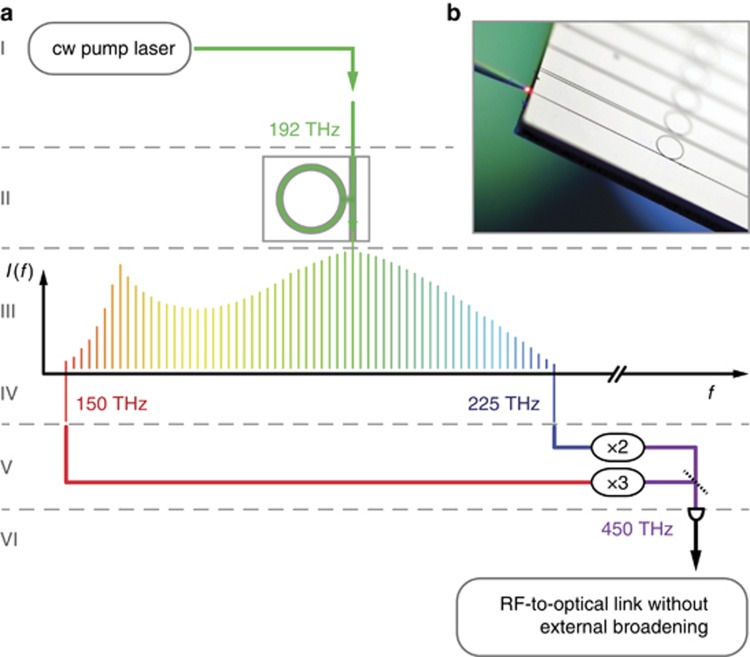
Schematic illustration of a self-referenced Kerr frequency comb. (**a**) The soliton Kerr frequency comb is generated from a continuous wave laser (I) coupled to a silicon nitride microresonator (II). With a spectral width of two-thirds of an octave, the resulting Kerr frequency comb (III) is sufficiently broad to allow for the measurement of its offset frequency (that is, the carrier envelope offset frequency). To do so, two transfer lasers (IV) are phase-locked to the frequency comb and doubled and tripled in frequency via nonlinear crystals (V). The heterodyne beat note of the two lasers allows the measurement of the offset frequency (*f*_CEO_) of the Kerr frequency comb (VI). (**b**) A photograph of a chip with integrated silicon nitride microresonators (240 μm diameter) and bus waveguides. Also shown is a lensed fiber used to couple light (in this photograph a red laser) into the waveguides on the chip.

Since the spectral span of our soliton frequency comb is two-thirds of an octave, the 2*f–*3*f* scheme (Figure 1a) can be applied. As with similar (*n*−1)*f*−*nf* schemes^6, 39^, the 2*f*–3*f* approach is a trade-off between optical bandwidth and the requirement of more complex nonlinear conversion. While for the common *f*–2*f* scheme a full octave of optical bandwidth but only one frequency doubling is required, the 2*f–*3*f* scheme requires only two-thirds of an octave but one frequency doubling and one frequency tripling. The resulting beat note after the nonlinear conversion is given by 3(*mf*_rep_+*f*_CEO_)*−*2(*nf*_rep_+*f*_CEO_)=*f*_CEO_ if 2*n*=3*m* (here *n* denotes the line number of the doubled frequency comb line and *m* the line number of the tripled line) and therefore enabling the measurement of the carrier envelope offset frequency. In order to achieve a sufficient signal-to-noise ratio, we implement the doubling and tripling stages using two transfer lasers that are phase locked to the Kerr frequency comb.

**Figure 2:**
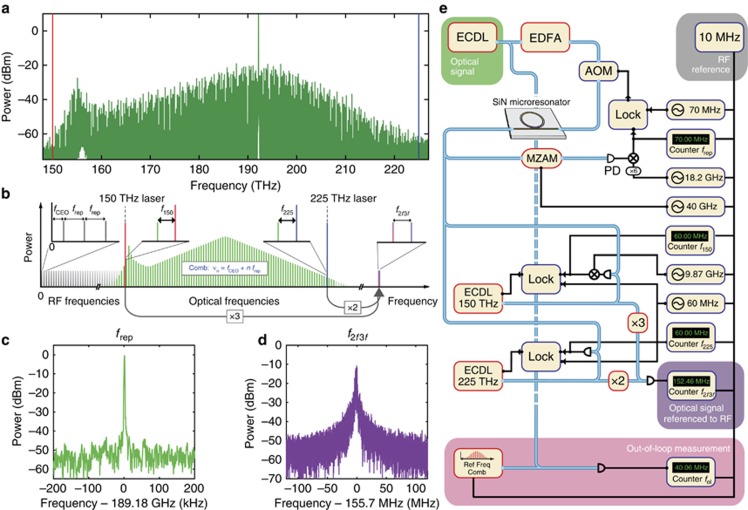
Setup and employed scheme for self-referencing of a soliton Kerr frequency comb. (**a**) Optical spectrum of the four-soliton state that was self-referenced in this work. (**b**) Schematic of the 2*f–*3*f* self-referencing with two transfer lasers as it was used in this work including the two stabilized frequency offsets *f*_150_ and *f*_225_ of the transfer lasers. (**c**) In-loop beat note of the stabilized repetition rate centered at 189.2 GHz. (**d**) Measurement of the free-running *f*_2*f*3*f*_ beat note that allows the computation of the offset frequency of the Kerr frequency comb. (**e**) Setup used for the experiments. AOM, acousto-optic modulator; ECDL, external cavity diode laser; EDFA, erbium-doped fiber amplifier; LOCK, combined phase comparator and proportional-integral-derivative (PID) servo controller for the phase locks; MZAM, Mach–Zehnder amplitude modulator; PD, photodiode. All RF frequencies are derived from one RF reference that is also used to reference the measurements. Blue lines represent optical fibers, black lines represent electrical connections.

The microresonator used in this work is a silicon nitride waveguide resonator^17, 40^ with a diameter of ∼240 μm (Figure 1b), resulting in a free spectral range of ∼190 GHz. It is pumped with an amplified external cavity diode laser operating at *ν*_pump_≈192.2 THz (1560 nm) that is coupled into the chip (∼2 W of continuous wave (cw) power in the waveguide, coupling efficiency around 50% per facet). The pumped fundamental TM mode family has an average linewidth of around 300 MHz corresponding to a loaded quality factor of 0.6 × 10^6^ which results in a measured threshold power in the bus waveguide of 300 mW^27^. The measured dispersion parameters^41^ are *D*_2_/2*π*:2.55 MHz, *D*_3_/2*π*:22.6 kHz and *D*_4_/2*π*:–173 Hz. Using the ‘power-kicking’ method^27^, the microresonator is brought into a soliton state that gives us the required bandwidth of two-thirds of an octave directly from the chip (Figure 2a). In this work, we used a four-soliton state as it could be generated reliable and had sufficient optical power. The two transfer lasers at ∼150 and ∼225 THz (2000 and 1330 nm, respectively) are phase-locked independently with frequency offsets of *f*_150_ and *f*_225_ to their nearest comb line (Figure 2b). The one transfer laser is then tripled in frequency (via second harmonic generation followed by sum frequency generation) while the other is doubled in frequency such that both have a frequency of around 450 THz (666 nm) where they generate the desired 2*f*–3*f* heterodyne beat note (*f*_2*f*3*f*_, Figure 2d).

Although the large line spacing of our frequency comb of *f*_rep_=189.184 GHz has the advantage that it can be easily resolved on an optical spectrometer, one challenge of the large mode spacing is that the measurement of the repetition rate as well as of the offset frequency requires the use of high-frequency photodiodes and RF components. In our experiment the repetition rate is measured via optical amplitude modulation down-mixing^42^ and RF down-mixing as shown in Figure 2c and 2e. We also take advantage of special properties of the 2*f–*3*f* scheme with transfer lasers to decrease the measured frequency *f*_2*f*3*f*_. First, it is important to note that with the 2*f–*3*f* scheme not all pairs of lines that are doubled and tripled, respectively, produce the same *f*_2*f*3*f*_ frequency. There are two relevant scenarios. The first one is if the condition 2*n*=3*m* is fulfilled as described above, then the heterodyne beat note *f*_2*f*3*f*_ is equal to *f*_CEO_. The second scenario is if 2*n*=3*m*+1 is fulfilled, then *f*_2*f*3*f*_=*f*_CEO_*−f*_rep_ is measured. Therefore, a pair of lines is chosen to minimize the value of the measured frequency, which in our case is the second scenario with 2*n*=3*m*+1. Second, taking into account the two frequency offsets of the transfer lasers in our setup, the measured *f*_2*f*3*f*_ can be expressed as *f*_2*f*3*f*_=*f*_rep_*−f*_CEO_+2*f*_225_*−*3*f*_150_^32^. Therefore, we use *f*_150_≈9.87 GHz to reduce the measured 2*f–*3*f* beat to a frequency of the order of 100 MHz (Figure 2d). In the future, microresonators with additional electrical heaters could allow for tuning of the offset frequency to lower values such that it can be detected with lower bandwidth RF electronics^43, 44^.

All frequencies (the repetition rate, the frequency offsets of the two transfer lasers as well as the 2*f–*3*f* beat note) are simultaneously monitored on RF frequency counters. These counters as well as all other RF equipment and in particular the RF synthesizers for the required local oscillators are referenced to a common 10 MHz reference derived from a commercial atomic clock (Figure 2e). While this is in principle sufficient for self-referencing as the offset frequency and repetition rate of the frequency comb can be computed from the counter measurements^32^, the repetition rate was also stabilized. For this, we compare the repetition rate of the Kerr frequency comb to the RF reference and feedback onto the pump power^27, 45^ using an acousto-optic modulator (Figure 2e). While the theoretical feedback bandwidth of this scheme is around 1 MHz, the system limits the usable bandwidth to a lower value, which is still sufficient for a stable phase-lock. We record the overlapping Allan deviations of the three locked frequencies. This is implemented using two gapless Π-type counters for the frequencies *f*_rep_ and *f*_225_ as well as one Λ-type counter with dead time for *f*_150_.

## Results and discussion

**Figure 3:**
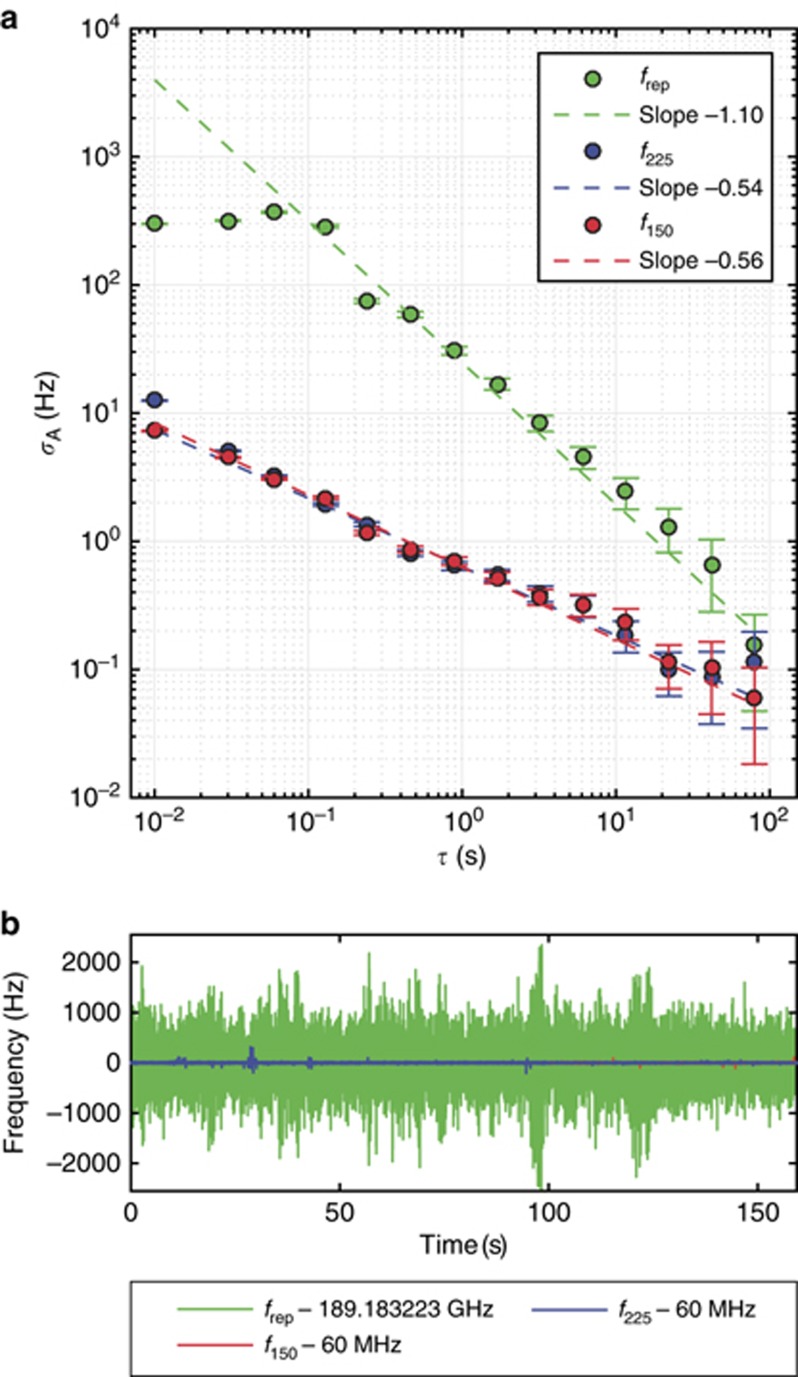
Counter measurements of the stabilized beat frequencies. (**a**) The overlapping Allan deviation *σ*_A_ of all locked frequencies (the repetition rate of the Kerr frequency comb *f*_rep_ and the offsets of the two transfer lasers *f*_150_ and *f*_225_) average down for longer gate times, showing all frequencies are indeed phase-locked. The flat part of the Allan deviation for *f*_rep_ is caused by the limited bandwidth of the actuation for this phase lock. The two transfer laser frequency offsets *f*_225_ and *f*_150_ are counted with different counters. The beat *f*_150_ is counted on a counter with dead time resulting in a slope of only around –0.5 instead of –1. The beat *f*_225_ is counted on a gapless counter. The reason for the deviation from the ideal *τ*^*−*1^ behavior for *f*_225_ are the occasional frequency excursions that the lock does not compensate perfectly. (**b**) The time trace of the locked and counted signals used to calculate the Allan deviation of **a**. The red trace is behind the blue trace. The locked repetition rate has much larger frequency excursions than the phase locks of the transfer lasers. Also visible are the excursions of *f*_225_ that cause the deviation from the ideal slope of –1 for the overlapping Allan deviation shown in **a**. The counter gate time for this data set is 10 ms.

As shown in Figure 3a, the overlapping Allan deviations of all three phase locks average down for longer timescales (gate times *τ>*0.1 s) indicating that the frequencies are indeed phase-locked. The flat behavior of the Allan deviation of *f*_rep_ for shorter timescales is due to the limited bandwidth of the actuation of its phase lock. However, the slope of −1.10 for longer time scales matches well the expected value of –1^46, 47^, showing that the phase lock compensates deviations on these timescales. In Figure 3b, the time traces of the frequency counters are displayed where the trace of the 225 THz transfer laser shows some smaller excursions. This results in the slope of –0.54 instead of –1 for this lock in the overlapping Allan deviation plot. The similar slope of the overlapping Allan deviation of the 150 THz laser lock however is mainly due to the different type of counter, which results in a slope close to the expected –0.5 for this Allan deviation^46, 47^. With an independent set of measurements we verify that these Allan deviations are not limited by the involved RF instruments. For example, the measured Allan deviation of around 5 × 10^−3^ at 1 s gate time for the 70 MHz frequency offset is well below the values shown in Figure 3a. Having phase-locked all frequencies but the offset frequency of the Kerr frequency comb, we can determine the value of the offset frequency as *f*_CEO_=*f*_rep_*−*3*f*_150_+2*f*_225_−*f*_2*f*3*f*_≈159.71 GHz and measure its drift by monitoring *f*_2*f*3*f*_.

**Figure 4:**
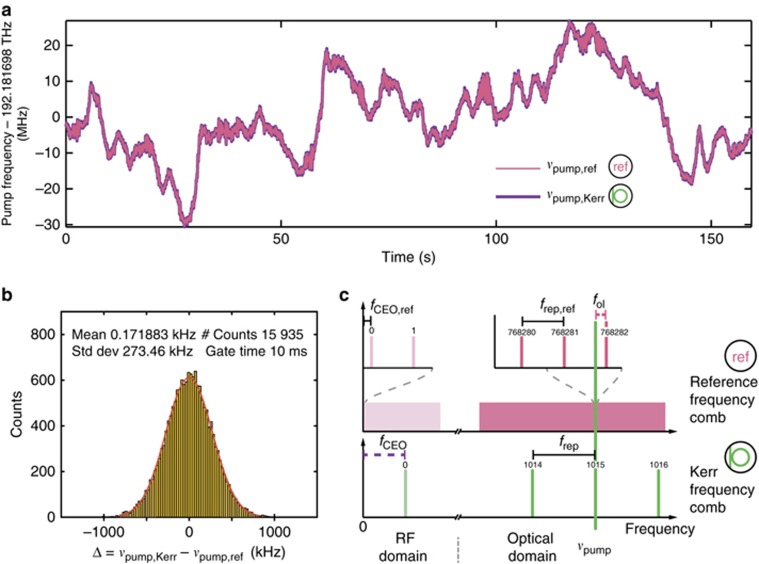
Tracking of the pump laser frequency with the self-referenced soliton Kerr frequency comb and an out-of-loop verification. (**a**) The drift of the frequency of the pump laser (*ν*_pump_) over time as measured with the Kerr frequency comb (thick, dark violet line, *ν*_pump,Kerr_=*f*_CEO_+1015*f*_rep_) and with a reference fiber frequency comb (thin, light pink line, *ν*_pump,ref_=*f*_CEO,ref_ + 768282*f*_rep,ref_*−f*_ol_). Gate time is 10 ms. As the repetition rate of the soliton Kerr frequency comb is locked and the pump laser itself is one line of the frequency comb, the drift of the pump laser is equivalent to the drift of the offset frequency of the Kerr frequency comb. The reference frequency comb is a fully self-referenced, stabilized fiber frequency comb. (**b**) The histogram of the difference of the two tracked pump laser frequencies Δ=*ν*_pump,Kerr_*−ν*_pump,ref_. The Gaussian fit (red) shows a deviation of 172 Hz from the expected mean of 0 Hz. (**c**) Illustration that shows all the frequencies involved in this out-of-loop measurement. Solid, black horizontal bars indicate locked frequencies. The two pink and violet dashed bars are the two frequencies that are not stabilized but counted in order to derive the data shown in **a** and **b**.

One unique property of Kerr frequency combs is that the pump laser constitutes one of the lines of the frequency comb. Because the repetition rate of our frequency comb is locked, the drift of the pump laser frequency is directly mapped to the excursion of the offset frequency of the frequency comb. Therefore the self-referenced Kerr frequency comb can be used to derive the exact optical frequency of the cw pump laser and to track it. This is confirmed by taking an out-of-loop measurement. For this experiment a fraction of the pump laser is split off before the microresonator and the heterodyne beat note of the pump laser with a commercial self-referenced, fully stabilized frequency comb (*f*_CEO,ref_=20 MHz, *f*_rep,ref_=250.14 MHz) is counted (Figure 2e). At the same time, the measured *f*_2*f*3*f*_ is counted as well (Figure 4c). The two frequency counters used are the same model of Π-type counters mentioned above and the reference frequency comb is stabilized to the same commercial atomic clock RF reference as all other RF equipment used in this experiment. By calculating the line number of the pump laser in the Kerr frequency comb (1015) and the line number of the line of the reference frequency comb that the pump laser beats with (768,282) and using our knowledge of all frequencies (*f*_rep_, *f*_CEO_, *f*_rep,ref_, *f*_CEO,ref_ and *f*_ol_) we can calculate the optical frequency of the pump laser in two ways. Once using the Kerr frequency comb and its counted offset frequency and once using the out-of-loop measurement with the commercial self-referenced fiber frequency comb. The overlay of these two independent frequency measurements over time is shown in Figure 4a. The correlation is very clear and no deviations are visible. In Figure 4b a histogram of the differences between the two optical frequencies is shown. The data fits well to a Gaussian distribution with the center frequency of the distribution shifted by 172 Hz from 0 Hz for the 160-s-long measurement. This out-of-loop experiment validates our ability to precisely determine the offset frequency of our Kerr frequency comb using the 2*f–*3*f* scheme.

## Conclusions

In summary, we demonstrate a self-referenced Kerr frequency comb without employing external broadening. Using dissipative Kerr soliton dynamics, we show that coupling a cw laser into an integrated, on-chip microresonator is enough to coherently ‘broaden’ its spectrum and to allow for self-referencing. Alleviating the need for additional external broadening in on-chip Kerr frequency comb devices shows that self-referenced, phase-stabilized integrated frequency comb sources are in principle possible. While transfer lasers are used in the current work, they do in principle constitute elements that are equally amenable to photonic integration^48^. Establishing devices that provide a microwave to optical link on a chip may catalyze a wide variety of applications such as integrated, microresonator-based atomic clocks^20^ and on-chip, low-noise RF synthesis from optical references^11^ and could contribute to making frequency metrology ubiquitous.
